# Single Nucleotide Polymorphism 8q24 rs13281615 and Risk of Breast Cancer: Meta-Analysis of More than 100,000 Cases

**DOI:** 10.1371/journal.pone.0060108

**Published:** 2013-04-02

**Authors:** Wen-Feng Gong, Jian-Hong Zhong, Bang-De Xiang, Liang Ma, Xue-Mei You, Qiu-Ming Zhang, Le-Qun Li

**Affiliations:** 1 Surgical Oncology Department, Tumor Hospital of Guangxi Medical University, Nanning, P. R. China; 2 Ward Senior Department, Tenth Affiliated Hospital of Guangxi Medical University, Qinzhou, P. R. China; AMS Biotechnology, United Kingdom

## Abstract

**Background:**

The onset and progression of breast cancer (BC) is influenced by many factors, including the single nucleotide polymorphism (SNP) rs13281615 at 8q24. However, studies of the potential association between rs13281615 at 8q24 and risk of BC have given inconsistent results. We performed a meta-analysis to address this controversy.

**Methods:**

PubMed, EMBASE and the Chinese National Knowledge Infrastructure databases were systematically searched to identify relevant studies. Two curators independently extracted data, and odds ratios (ORs) with 95% confidence intervals (95% CIs) were calculated to assess the strength of the association between rs13281615 at 8q24 and risk of BC.

**Results:**

Fourteen studies are included in the meta-analysis, involving 44,283 cases (5,170 Chinese and 39,113 mixed) and 55,756 controls (5,589 Chinese and 50,167 mixed). The GG and G-allele genotypes of rs13281615 at 8q24 are significantly associated with increased risk of BC (GG vs. AG+AA, OR 1.13, 95% CI 1.08–1.19, *P*<0.001; G-allele vs. A-allele, OR 1.10, 95% CI 1.06–1.14, *P*<0.001; GG vs. AA, OR 1.20, 95% CI 1.12–1.29, *P*<0.001). Conversely, the AA genotype is significantly associated with decreased risk of BC (AA vs. AG+GG, OR 0.89, 95% CI 0.84–0.93, *P*<0.001).

**Conclusion:**

G-allele genotypes of rs13281615 at 8q24 polymorphism are a risk factor for developing BC, while the AA genotype is a protective factor. Further large and well-designed studies are required to confirm this conclusion.

## Introduction

Breast cancer (BC) is one of the most prevalent invasive cancers and the second leading global cause of cancer-related deaths among women [1]. Global BC incidence has been increasing by more than one million new cases annually [2]. Incidence is significantly higher in developed countries than in developing ones; in fact, it was the first malignant disease to pose a significant threat to women [3], [4]. Despite the frequency and severity of BC, the pathogenesis and progression of BC are still not fully understood.

Many researchers have concluded that BC is the cumulative result of multiple environmental factors and genetic alterations [5]. Epidemiological studies have suggested that estrogen stimulation [6], high birth weight [7], obesity [8] and family history of BC [9], [10] may be associated with increased risk of BC in postmenopausal women. However, only some patients with family history of BC develop malignancy; most cases of BC are sporadic. Therefore, genetic polymorphism may contribute to the development of BC. This is supported by numerous meta-analyses that have found certain genetic polymorphisms to correlate strongly with susceptibility to BC [11]–[13]. High-penetrance breast cancer susceptibility genes, such as *BRCA1* and *BRCA2*, have low mutation rates and therefore explain only a small fraction of breast cancers in the general population [14]. This suggests the need to identify additional polymorphisms linked to risk of BC.

Two large-scale genome-wide association studies identified several common polymorphisms that may be linked to susceptibility to BC [15], [16], including single nucleotide polymorphisms (SNPs) at rs 13281615. Located in the non-coding chromosomal region 8q24, the function of rs 13281615 is unclear. The locus lies near the myelocytomatosis oncogene (MYC) at 8q24.12–24.13, which can promote cell proliferation, differentiation and transformation. It plays an important role in the development of many types of cancer, including BC [17]. Sole and coworkers [18] proposed that variations in putative cis-regulators of transcription in 8q24 can significantly alter germline c-MYC expression levels and thereby contribute to cancer susceptibility.

Ever since three studies [15], [19], [20] reported associations between the 8q24 rs13281615 SNP and various cancers, including BC, numerous epidemiological studies have evaluated the association between rs13281615 at 8q24 and BC and have generated sometimes inconsistent results. To provide a clearer picture of the effects of this SNP on risk of BC, a meta-analysis was performed.

## Materials and Methods

### Publication Search Strategy

PubMed, EMBASE and the Chinese National Knowledge Infrastructure (CNKI) databases were searched through December 2012 for case-control studies about 8q24 rs13281615 SNP and BC risk. The following search terms were used: “8q24,” “rs13281615,” “SNP,” “single nucleotide polymorphism,” “polymorphism,” “mutation,” “variant,” “BC,” “breast neoplasm” and “breast cancer.” Reference lists in each identified article were also searched manually to identify additional eligible studies.

### Inclusion Criteria

To be included in the meta-analysis, studies had to (1) assess the association between the 8q24 rs13281615 SNP and risk of BC occurrence, (2) use a case-control design and (3) provide sufficient data for estimating odds ratios (ORs) with 95% confidence intervals (CIs). In the case of multiple studies by the same researchers involving the same or overlapping data sets, we selected the most recent study with the largest number of participants.

### Data Extraction

Two curators (W-FG and J-HZ) independently extracted information from included studies. Disagreement was resolved by discussion between the two authors. The following data were extracted: first author’s family name, year of publication, country of origin, source of controls, total numbers of cases and controls, Hardy-Weinberg equilibrium (HWE) of controls and the frequency of rs13281615 genotypes at 8q24 in cases and controls.

### Statistical Methods

All statistical tests were performed using Review Manager 5.1.4 software. The strength of association between the 8q24 rs13281615 SNP and BC risk was assessed by calculating ORs with 95% CIs based on the genotype frequencies in cases and controls. Subgroup analysis was performed based on ethnicity, categorized as Chinese or mixed (predominantly Caucasian).

The significance of pooled ORs was determined using the Z-test, with *P*<0.05 defined as the significance threshold. Meta-analysis was conducted using the random-effects model, except when *P*>0.10 for the Q-test, indicating lack of heterogeneity among studies; in this case, the fixed-effects model was used. Publication bias was assessed by visual inspection of Begg’s funnel plots. HWE in the control group was assessed using the asymptotic test, with *P*<0.05 considered significant.

## Results

### Description of Studies

**Figure 1:**
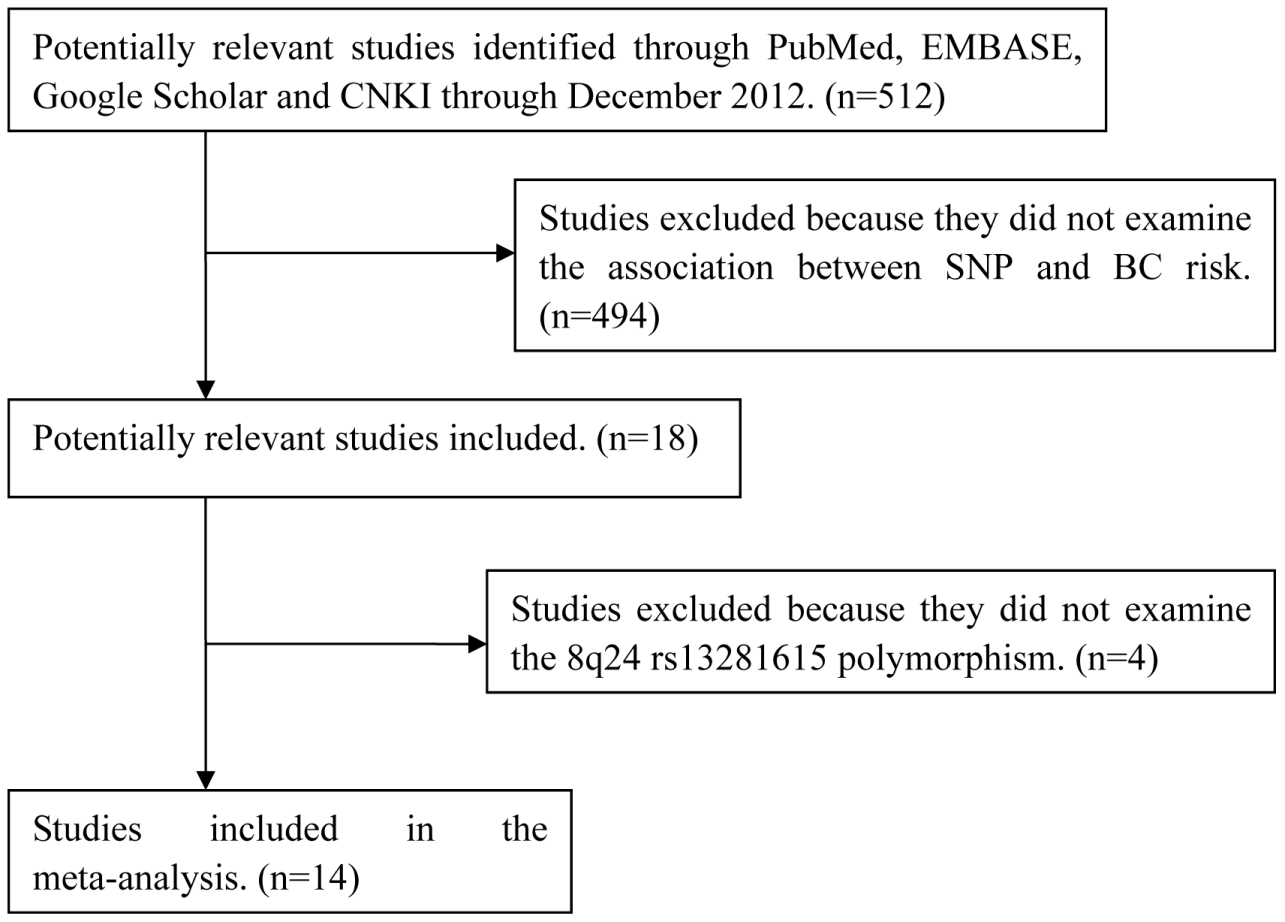
Flow chart of study selection. (CNKI, Chinese National Knowledge Infrastructure; SNP, single nucleotide polymorphism; BC, breast cancer.).

Database searches through December 2012 revealed 512 potentially relevant publications about rs13281615 at 8q24 and risk of BC (Figure 1). Screening titles and abstracts led to exclusion of 494 articles because they were laboratory studies or review articles, they examined other 8q24 polymorphisms, or they were irrelevant to the current study. This left 18 articles, which were read in full. In the end, 14 articles were found to satisfy the inclusion criteria and were included in the meta-analysis [21]–[34]. These articles involved a total of 44,283 cases and 55,756 controls, of which 5,170 cases and 5,589 controls were Chinese [25]–[27], [32], and the remaining 39,113 cases and 50,167 controls were of mixed ethnicity (>95% Caucasian) [21]–[24], [28]–[31], [33], [34]. In one study [22], 95.6% of cases and 96.7% of controls were European, while 4.4% of cases and 3.3% of controls were Asian. The distribution of rs13281615 genotypes at 8q24 in the controls was consistent with HWE (*P*>0.05) in all but three studies [24], [28], [29]. Table 1 shows principal characteristics of the included studies.

**Table 1:** Principal characteristics of studies included in the meta-analysis.

Study	Country or Ethnicity	Subjects		*P*_HWE_	Case genotypes			Control genotypes		
		Cases	Controls		AA	AG	GG	AA	AG	GG
Fletcher 2008^21^	Caucasian	Bilateral, family history	PB	0.37	435	730	305	487	629	225
Garcia-Closas 2008^22^	Caucasian and Asian	Sporadic	PB	0.67	4879	7284	2921	7650	10682	3773
Mcinerney 2009^23^	Caucasian	Sporadic	PB	0.10	272	467	178	355	456	182
Tamimi 2010^24^	Caucasian	Sporadic	PB	<0.001	175	263	223	161	277	273
Long 2010^25^	Chinese	Sporadic	PB	0.99	679	1470	796	745	1491	745
Li 2011^26^	Chinese	Sporadic	HB	0.75	111	285	162	149	313	173
Jiang 2011^27^	Chinese	Sporadic	PB	1.00	121	247	125	128	255	127
Harlid 2012^28^	Caucasian	Sporadic	PB	0.04	1103	1723	719	1766	2357	884
Teraoka 2011^29^	Caucasian	Sporadic	PB	0.04	174	292	140	358	623	213
Antoniou 2009^30^	Caucasian	Sporadic	PB	0.10	2519	3872	1396	2187	3317	1158
Gorodnova 2010^31^	Caucasian	Sporadic	PB	0.71	35	63	42	52	84	38
Chan 2012^32^	Chinese	Sporadic	PB	0.05	303	554	317	406	693	364
Campa 2011^33^	Caucasian	Sporadic	PB	0.11	2494	4044	1764	3813	5609	2193
Shan 2012^34^	Tunisians	Sporadic	PB	0.50	126	281	194	93	176	96

Note: HWE, Hardy-Weinberg equilibrium; PB, population-based; HB, hospital-based.

### Test of Heterogeneity

Table 2 shows the association between the 8q24 rs13281615 SNP and BC risk. The heterogeneity of 8q24 rs13281615 A/G allelic contrast, homozygote comparison, and dominant and recessive genetic models was analyzed for all 14 studies. Random-effects models were used to meta-analyze the entire population and the mixed population. The fixed-effects model was used to meta-analyze the Chinese population.

**Table 2:** Overall and stratified meta-analyses of the association between the 8q24 rs13281615 single nucleotide polymorphism and breast cancer risk.

Genotype comparison	OR [95% CI]	Z (*P* value)	Heterogeneity of study design				Model
			?^2^		df (*P* value)	I^2^	
**Total (44,283 cases, 55,756 controls)**							
G-allele vs. A-allele	1.10 [1.06, 1.14]	4.77 (<0.001)		39.07	13 (<0.001)	67%	Random
GG vs. AA	1.20 [1.16, 1.24]	9.80 (<0.001)		29.50	13 (0.006)	56%	Random
GG vs. AG+AA	1.13 [1.08, 1.19]	4.87 (<0.001)		22.54	13 (0.05)	42%	Random
AG+GG vs. AA	1.13 [1.07, 1.19]	4.76 (<0.001)		26.71	13 (0.01)	51%	Random
AA vs. AG+GG	0.89 [0.84, 0.93]	4.76 (<0.001)		26.71	13 (0.01)	51%	Random
**Ethnic subgroups**							
**Chinese Population (5,170 cases, 5,589 controls)**							
G-allele vs. A-allele	1.07 [1.02, 1.13]	2.57 (0.01)		2.30	3 (0.51)	0%	Fixed
GG vs. AA	1.17 [1.05, 1.30]	2.84 (0. 005)		0.61	3 (0.89)	0%	Fixed
GG vs. AG+AA	1.10 [1.01, 1.20]	2.22 (0.03)		0.30	3 (0.96)	0%	Fixed
AG+GG vs. AA	1.11 [1.02, 1.22]	2.39 (0.02)		0.83	3 (0.84)	0%	Fixed
AA vs. AG+GG	0.90 [0.82, 0.98]	2.39(0.02)		0.83	3 (0.84)	0%	Fixed
**Mixed population (39,113 cases, 50,167 controls)**							
G-allele vs. A-allele	1.11 [1.06, 1.17]	4.29 (<0.001)		36.16	9 (<0.001)	75%	Random
GG vs. AA	1.22 [1.12, 1.33]	4.40 (<0.001)		28.62	9 (<0.001)	69%	Random
GG vs. AG+AA	1.15 [1.07, 1.22]	4.04 (<0.001)		21.59	9 (0.01)	58%	Random
AG+GG vs. AA	1.13 [1.07, 1.21]	3.94 (<0.001)		25.88	9 (0.002)	65%	Random
AA vs. AG+GG	0.88 [0.83, 0.94]	3.94 (<0.001)		25.88	9 (0.002)	65%	Random

### Quantitative Data Synthesis

Table 2 shows the summary ORs relating the 8q24 rs13281615 SNP to BC risk based on 44,283 cases and 55,756 controls in all 14 studies. We observed an association between rs13281615 genotype at 8q24 and BC risk in the total population, the mixed population and the Chinese population.

#### Total population

**Figure 2:**
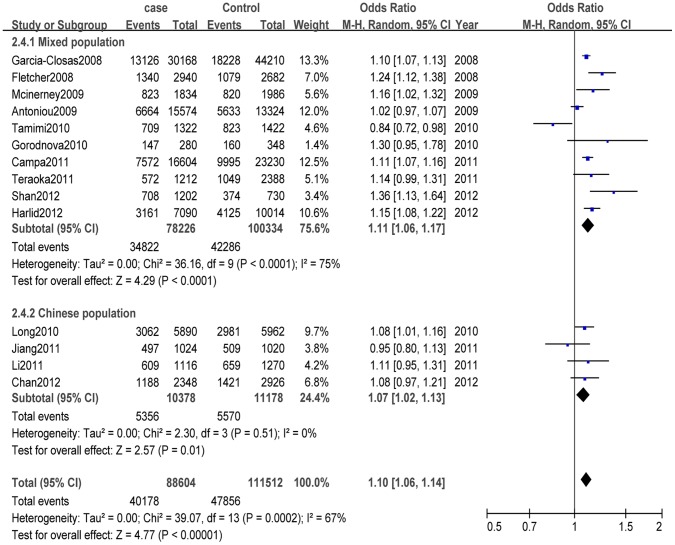
Forest plots describing the association of the 8q24 rs13281615 single nucleotide polymorphism with risk of developing breast cancer (G-allele vs. A-allele).

When the dominant genetic comparison model was applied to genotype data for the total population, individuals with the AA genotype of rs13281615 at 8q24 were found to have lower BC risk than individuals with other genotypes based on the random-effects model (OR 0.89, 95% CI 0.84–0.93, *P<*0.001; I*^2^* = 51%) (Figure S1). In the recessive comparison model, participants with a GG genotype had a higher risk of BC (OR 1.13, 95% CI 1.08–1.19, *P<*0.001; I^2^ = 42%) (Figure S2). In the homozygote comparison model, the GG genotype was also associated with higher risk of BC, with a pooled OR of 1.20 (95% CI 1.12–1.29, *P<*0.001; I^2^ = 56%) (Figure S3). Most important, by allelic comparison, G-allele genotypes of rs13281615 at 8q24 were associated with higher risk of BC, with a pooled OR of 1.10 (95% CI 1.06–1.14, *P<*0.001; I^2^ = 67%) (Figure 2).

#### Mixed population

After stratifying the data by ethnicity and applying the recessive comparison model to the mixed population, we found the GG genotype to be associated with higher risk of BC (OR 1.15, 95% CI 1.07–1.22, *P<*0.001, I^2^ = 58%). In the homozygote comparison model, the GG genotype was associated with higher risk of BC (OR 1.22, 95% CI 1.12–1.33, *P<*0.001, I^2^ = 69%). In the dominant genetic comparison model, the AA genotype was associated with lower risk of BC (OR 0.88, 95% CI 0.83–0.94, *P<*0.001, I^2^ = 65%). By allelic comparison, the G-allele was also associated with lower risk of BC (OR 1.11, 95% CI 1.06–1.17, *P<*0.001, I^2^ = 75%).

#### Chinese population

Analysis of the Chinese population in four studies [25]–[27], [32] revealed that the GG genotype was associated with higher risk of BC based on the fixed-effects model (GG vs. AA, OR 1.17, 95% CI 1.05–1.30, *P* = 0.005, I^2^ = 0%; GG vs. AG+AA, OR 1.10, 95% CI 1.01–1.20, *P* = 0.03, I^2^ = 0%; G-allele vs. A-allele, OR 1.07, 95% CI 1.02–1.13, *P* = 0.01, I^2^ = 0%). The AA genotype was associated with lower risk of BC based on the random-effects model (AA vs. AG+GG, OR 0.90, 95% CI 0.82–0.98, *P* = 0.02, I^2^ = 0%).

### Sensitivity Analysis

Sensitivity analysis was carried out by excluding one study with large heterogeneity [24] or another study with a large weight [22]. In neither case were the pooled ORs significantly affected (data not shown), implying that our results based on all 14 studies were robust. Sensitivity analysis was done by using the random-effect model or the fixed-effect model. Results were not altered (Table S1).

### Publication Bias

Begg’s funnel plots were calculated to assess publication bias for reported comparisons of 8q24 rs13281615 SNP and risk of BC. The shape of the funnel plot seemed asymmetrical for GG vs. AG+AA, AA vs. AG+AA, GG vs. AA, and G-allele vs. A-allele, suggesting the presence of publication bias (Figure S4).

## Discussion

As with other malignancies, the pathogenesis of BC involves environmental factors, molecular signaling pathways and host genetic factors. Genome-wide association studies conducted since 2007 have identified several genetic loci associated with susceptibility to BC. Some studies have reported an association between 8q24 rs13281615 SNP and BC risk, while others have found no such association. The most likely reason for the inconsistencies among these studies is that they involve relatively small samples. To conduct association studies with larger numbers of participants, we conducted a meta-analysis of published studies. Our results for the total population suggest increased BC risk for subjects carrying the G-allele of rs13281615 at 8q24.

Subgroup analysis by ethnicity allowed us to look for potential ethnic differences in the association. In the Chinese population, the G-allele was associated with increased risk of BC based on allelic contrast, recessive contrast and homozygote comparison. Similarly, for the mixed population (>95% Caucasian), the G-allele was also associated with increased risk of BC. In both the Chinese and mixed populations, the AA genotype of rs13281615 was associated with lower risk of BC. Sensitivity analysis did not alter these results.

Several studies included in the meta-analysis demonstrate that rs13281615 polymorphisms can interact with environmental factors to modulate BC risk [23]–[27], [29], [32], [34]. In fact, rs13281615 polymorphisms were not found to interact with BRCA1 or BRCA2 in BC risk, though only one study [30] examined the possibility of such genetic interactions. These results indicate that the etiology of BC is complex and involves host and environmental factors that may interact synergistically. Indeed, growing epidemiologic evidence suggests that different types of BC have different risk factor profiles and may therefore occur via different pathways.

Incidence of BC and associated mortality differ widely across ethnicities [35]. Caucasians ancestry increases the risk of BC [36]. Incidence of BC among women in most North American and European countries is more than triple that in Asian countries [37]. After analyzing various genetic loci in BC patients in Europe, North America, Australia and Southeast Asia, Easton et al. [15] also suggested that the frequency of the G-allele of rs13281615 at 8q24 was significantly higher among Caucasians than among other populations. Our meta-analysis gave similar results: the G-allele of rs13281615 at 8q24 was a risk factor for BC in the mixed population (>95% Caucasian). At the same time, the G-allele was a risk factor in the Chinese population, which surprisingly showed a higher frequency of the G-allele than did the mixed population. This discrepancy from the work of Easton et al. [15] may reflect the complex multifactorial etiology of BC.

There are several limitations in this meta-analysis. First, the controls were not uniformly defined. Although all controls were healthy populations, it is possible that some of them were community-based, while others were hospital-based. The *P* value of HWE of three included studies [24], [28], [29] was less than 0.05 (Table 1), suggesting that these study populations were not representative of the broader target population. Second, BC onset and progression are affected by multiple factors, but because of lacking of detailed data, we were unable to conduct stratified analyses based on possible confounders such as age, hormone level, or age at menarche and menopause. Therefore our data may have lacked sufficient statistical power to detect certain associations. Third, again because of a lack of detailed data, we could not examine interactions between the 8q24 rs13281615 SNP and environmental factors or genes known to affect risk of BC. Many gene polymorphisms are associated with risk of BC, and it may be that any single SNP such as rs13281615 at 8q24 is insufficient to cause BC on its own.

Our meta-analysis suggests that the 8q24 rs13281615 SNP affects risk of developing BC: the G-allele increases susceptibility to BC, while the A-allele protects against it. These findings add BC to the list of diseases for which the 8q24 rs13281615 SNP has been implicated as a susceptibility factor, together with prostate cancer [19] and colorectal cancer [11]. Further detailed investigation involving large, multiethnic samples is needed to clarify the role of this SNP in BC, as well as explore gene-gene and gene-environment interactions that may mediate the association between rs13281615 and BC risk.

This meta-analysis is guided by the PRISMA statement (Checklist S1).

## Supporting Information

Figure S1 Forest plots describing the association of the 8q24 rs13281615 single nucleotide polymorphism with risk of developing breast cancer (AA vs. AG+GG).
(TIF)

Figure S2 Forest plots describing the association of the 8q24 rs13281615 single nucleotide polymorphism with risk of developing breast cancer (GG vs. AG+AA).
(TIF)

Figure S3 Forest plots describing the association of the 8q24 rs13281615 single nucleotide polymorphism with risk of developing breast cancer (GG vs. AA).
(TIF)

Figure S4 Begg’s funnel plots to examine publication bias for reported comparisons of the 8q24 rs13281615 single nucleotide polymorphism and risk of BC. Data were plotted using pseudo 95% confidence limits. SE, standard error.
(TIF)

Table S1 Sensitivity analysis results with random-effect model and fixed-effect model.
(DOC)

Checklist S1 PRISMA 2009 Checklist.
(DOC)
