# Supplementary figures and images for: Single Nucleotide Polymorphism 8q24 rs13281615 and Risk of Breast Cancer: Meta-Analysis of More than 100,000 Cases
Source: PLoS One. 2013 Apr 2;8(4):e60108. doi: 10.1371/journal.pone.0060108 (PMC3614948; doi:10.1371/journal.pone.0060108)

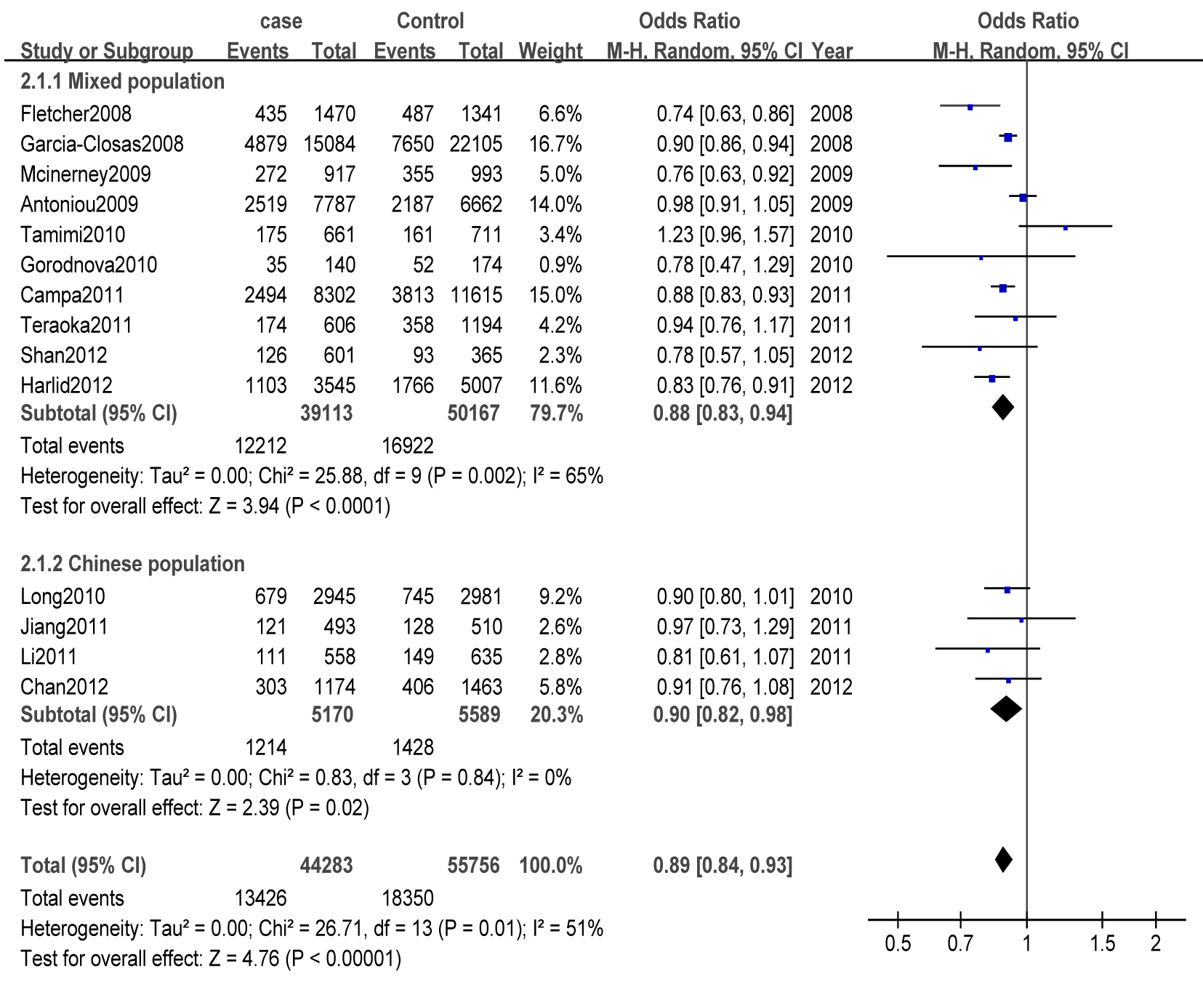

Supplement: Figure S1 — Forest plots describing the association of the 8q24 rs13281615 single nucleotide polymorphism with risk of developing breast cancer (AA vs. AG+GG). (TIF) [file pone.0060108.s001.tif]

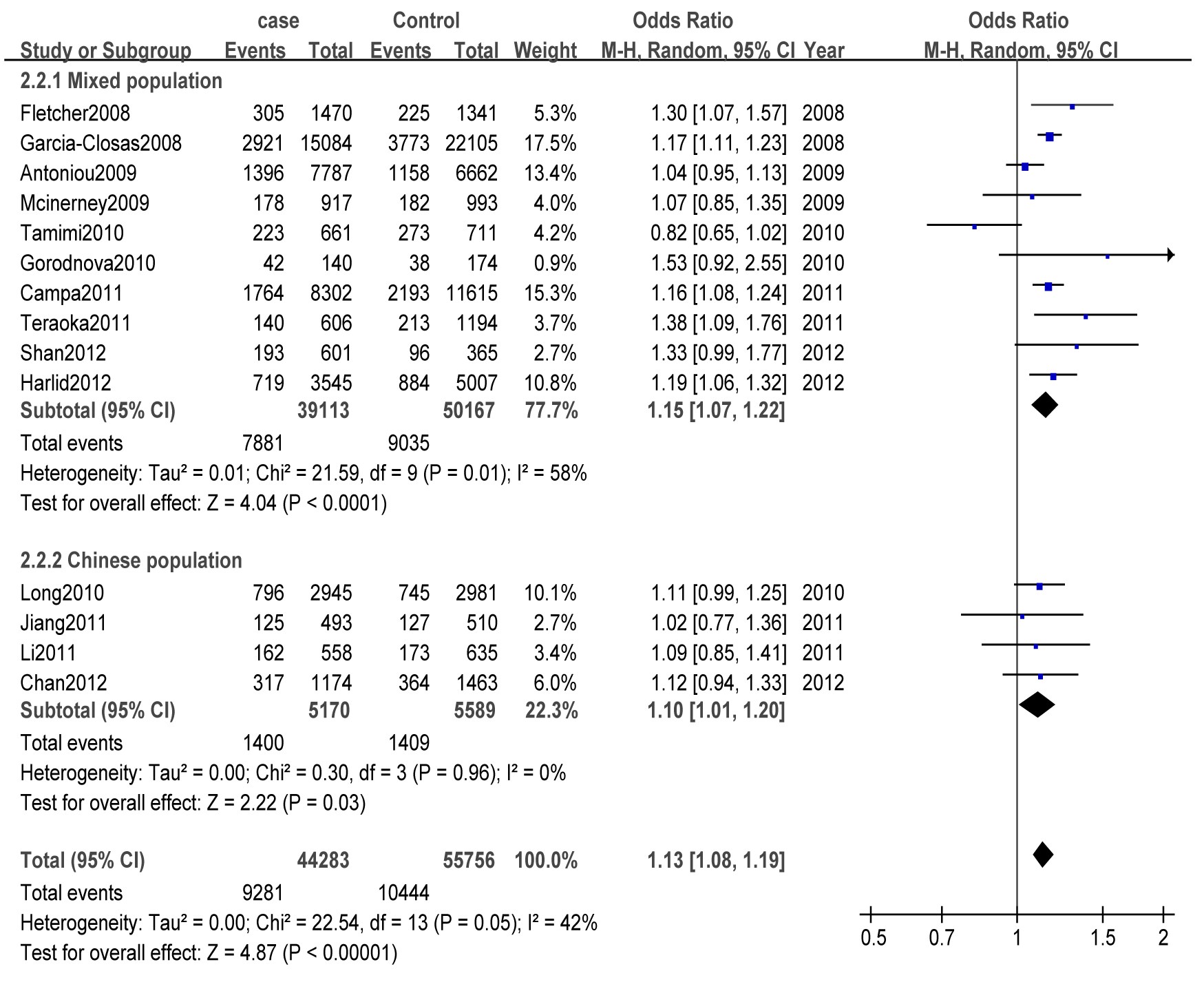

Supplement: Figure S2 — Forest plots describing the association of the 8q24 rs13281615 single nucleotide polymorphism with risk of developing breast cancer (GG vs. AG+AA). (TIF) [file pone.0060108.s002.tif]

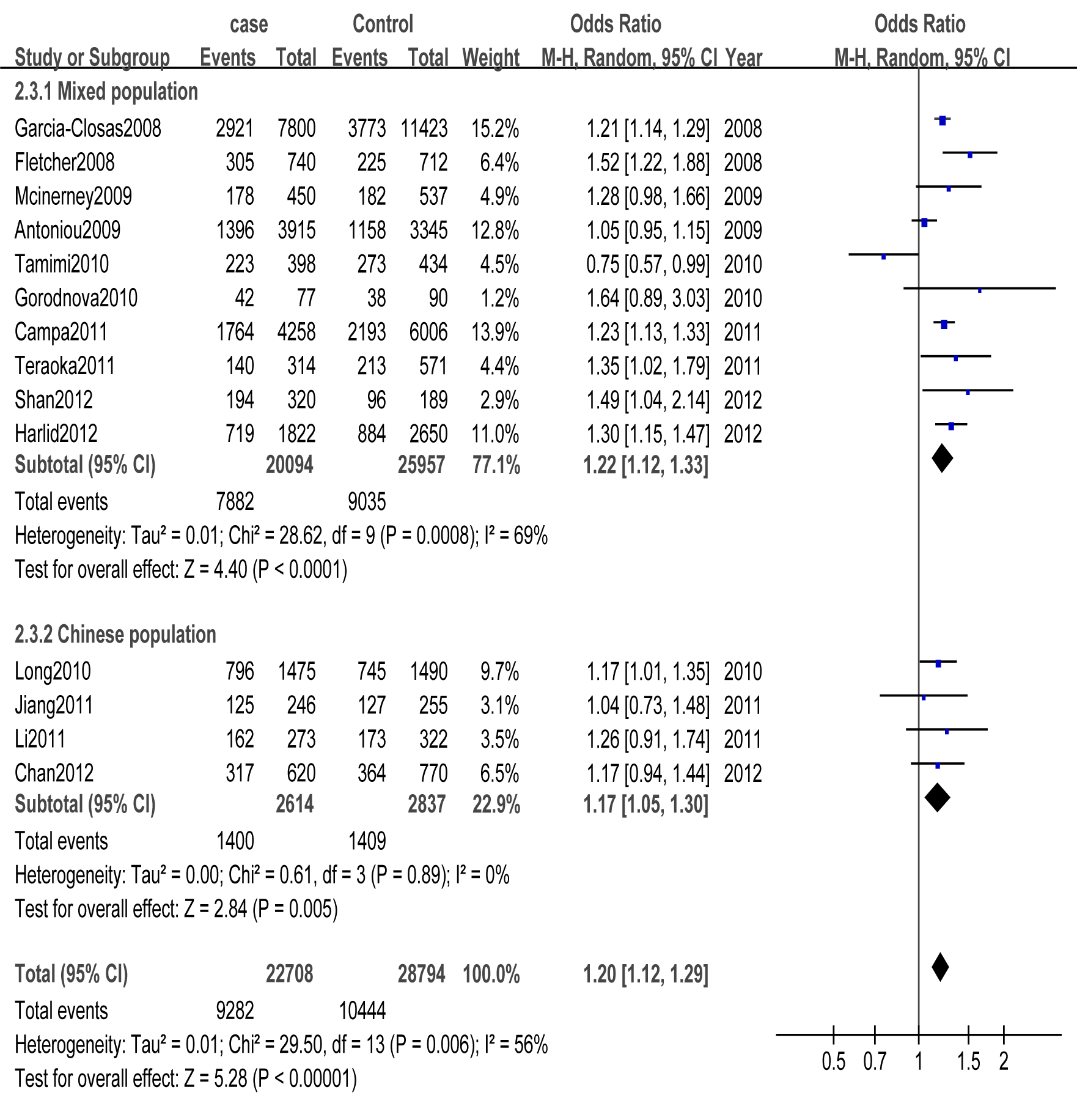

Supplement: Figure S3 — Forest plots describing the association of the 8q24 rs13281615 single nucleotide polymorphism with risk of developing breast cancer (GG vs. AA). (TIF) [file pone.0060108.s003.tif]

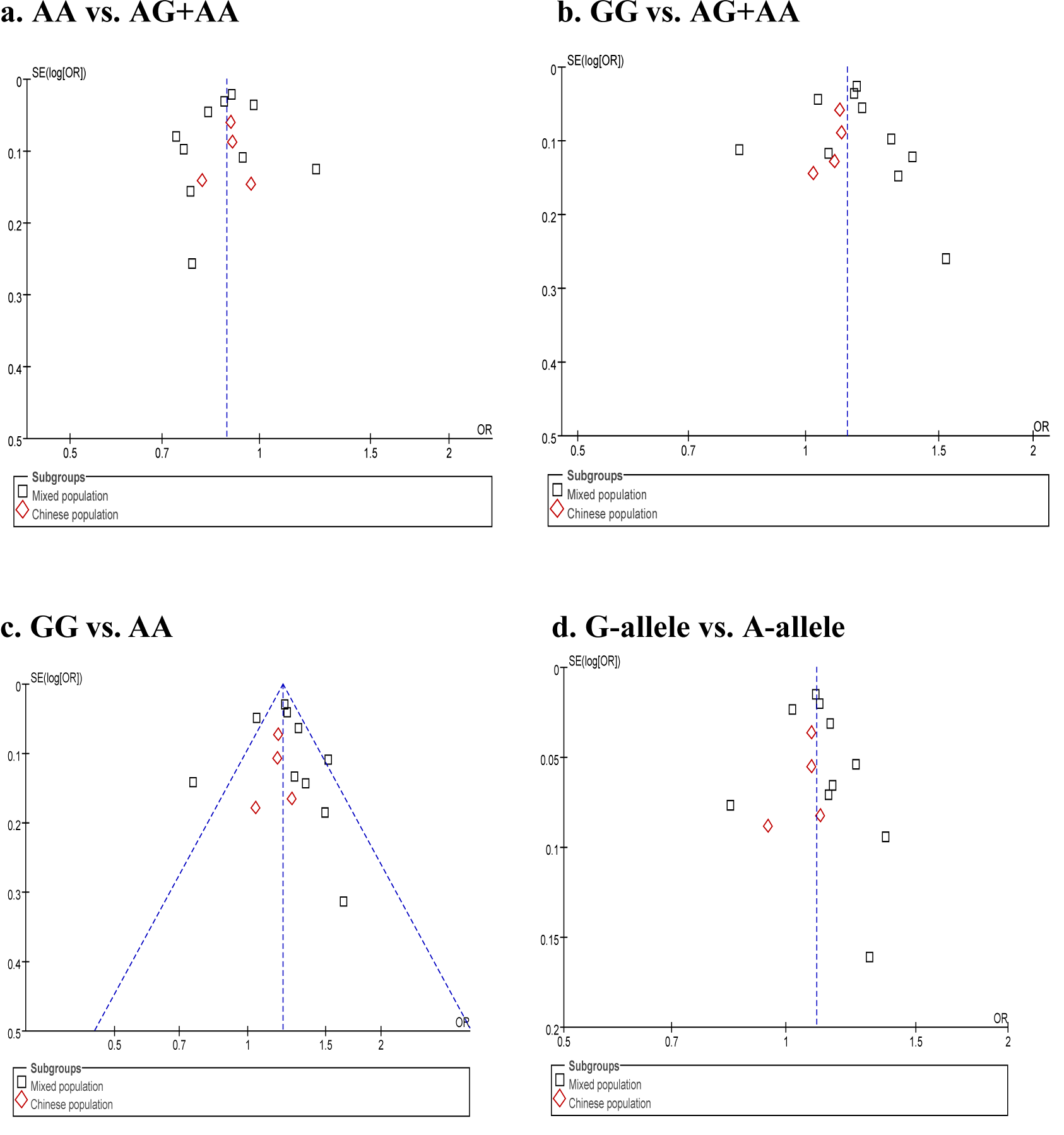

Supplement: Figure S4 — Begg’s funnel plots to examine publication bias for reported comparisons of the 8q24 rs13281615 single nucleotide polymorphism and risk of BC. Data were plotted using pseudo 95% confidence limits. SE, standard error. (TIF) [file pone.0060108.s004.tif]
