# Supplementary material for: Single Nucleotide Polymorphism 8q24 rs13281615 and Risk of Breast Cancer: Meta-Analysis of More than 100,000 Cases
Source: PLoS One. 2013 Apr 2;8(4):e60108. doi: 10.1371/journal.pone.0060108 (PMC3614948; doi:10.1371/journal.pone.0060108)
Supplement: Table S1 — Sensitivity analysis results with random-effect model and fixed-effect model. (DOC) [file pone.0060108.s005.doc]

Table S1. Sensitivity analysis for total population

| Genotype comparison | Fixed effect model | | Random effect model | |
| --- | --- | --- | --- | --- |
|  | OR(95%CI) | *P* value | OR(95%CI) | *P* value |
| G-allele vs. A-allele | 1.09 [1.08,1.11] | <0.001 | 1.10 [1.06,1.14] | <0.001 |
| GG vs. AG+AA | 1.14 [1.10,1.18] | <0.001 | 1.13 [1.08,1.19] | <0.001 |
| GG+AG vs. AA | 1.12 [1.09,1.15] | <0.001 | 1.13 [1.07,1.19] | <0.001 |
| GG vs. AA | 1.20 [1.16,1.24] | <0.001 | 1.20 [1.12,1.29] | <0.001 |
| AA vs. AG+GG | 0.90 [0.87,0.92] | <0.001 | 0.89 [0.84,0.93] | <0.001 |
